# ID 234 - Avaliação do Acesso a Medicamentos Incorporados pelo SUS para Doenças Raras

**DOI:** 10.5327/2237-9622.2025.v34s1.234

**Published:** 2025-11-25

**Authors:** Silvana Marcia Bruschi Kelles, Silvana Marcia Bruschi Kelles, Marcus Carvalho Borin, Mariana Michel Barbosa, Carina Rejane Martins, Daniel Pitchon dos Reis, Geraldo José Coelho Ribeiro, Júlia Teixeira Tupinambás, Karina de Castro Zocrato, Lélia Maria de Almeida Carvalho, Marcela Pinto de Freitas, Maria da Glória Cruvinel Horta, Mariza Cristina Torres Talim, Sergio Adriano Loureiro Bersan

**Keywords:** Conitec, SUS, ANS, acesso a medicamentos

## Abstract

**Introdução:**

A integralidade do Sistema Único de Saúde (SUS), conforme a Lei Orgânica da Saúde (Lei n.º 8.080, de 19 de setembro de 1990), garante o acesso a tecnologias de saúde, incluindo medicamentos. A partir da criação da Comissão Nacional de Incorporação de Tecnologias no SUS (Conitec), em 2011, houve mais agilidade e transparência no processo de incorporação de tecnologias. Doenças raras representam um desafio significativo em termos de acesso a medicamentos, tanto no setor público quanto no privado. Este estudo avalia as incorporações de medicamentos para doenças raras pelo SUS entre 2019 e 2024, comparando o acesso entre os sistemas público e privado.

**Método:**

Realizou-se um estudo descritivo e exploratório por meio da análise quantitativa das incorporações de medicamentos recomendadas pela Conitec entre janeiro de 2019 e março de 2024. A doença rara é aquela que acomete até 65 pessoas por 100 mil indivíduos, segundo a Organização Mundial da Saúde (OMS). A aquisição do medicamento foi pesquisada no Banco de Preços em Saúde (BPS) via Sistema Integrado de Administração de Serviços Gerais (Siasg). Considerou-se medicamento de alto custo aquele cujo custo unitário excede 70% do salário mínimo.

**Resultados:**

Entre 2019 e 2024, a Conitec recomendou 151 incorporações, das quais 51 (33,8%) referiam-se a doenças raras. Dessas 51, 43 eram medicamentos, 34 (79,1%) deles sendo de alto custo e destinados a uma única indicação. No entanto, cinco (14,7%) desses medicamentos não apresentaram registro de compra no BPS nos últimos dois anos, indicando que, apesar da incorporação, os pacientes não estão tendo o acesso esperado. Além disso, para seis (17,6%) medicamentos com compra registrada no sistema, a quantidade adquirida era insuficiente para atender à demanda prevista para o tratamento.

**Conclusão:**

A análise revela uma disparidade significativa entre a incorporação de medicamentos para doenças raras e o acesso efetivo a eles no SUS. Esses resultados sugerem a necessidade de estratégias mais eficazes para garantir que a incorporação resulte em acesso real aos medicamentos para doenças raras no SUS.

